# Red blood cell-derived arginase release in hemolytic uremic syndrome

**DOI:** 10.1186/s12967-023-04824-x

**Published:** 2024-01-04

**Authors:** Niklas Friberg, Ida Arvidsson, Ashmita Tontanahal, Ann-Charlotte Kristoffersson, Magnus Gram, Bernard S. Kaplan, Diana Karpman

**Affiliations:** 1 Department of Pediatrics, Clinical Sciences Lund, Lund University, 221 85 Lund, Sweden; 2 Skåne University Hospital, Lund, Sweden; 3 Division of Nephrology, Children’s Hospital of Philadelphia, Philadelphia, PA USA

**Keywords:** Arginase, Hemolytic uremic syndrome, Thrombotic microangiopathy, Nitric oxide, Shiga toxin

## Abstract

**Background:**

Hemolysis is a cardinal feature of hemolytic uremic syndrome (HUS) and during hemolysis excess arginase 1 is released from red blood cells. Increased arginase activity leads to reduced L-arginine, as it is converted to urea and L-ornithine, and thereby reduced nitric oxide bioavailability, with secondary vascular injury. The objective of this study was to investigate arginase release in HUS patients and laboratory models and correlate arginase levels to hemolysis and kidney injury.

**Methods:**

Two separate cohorts of patients (n = 47 in total) with HUS associated with Shiga toxin-producing enterohemorrhagic *E. coli* (EHEC) and pediatric controls (n = 35) were investigated. Two mouse models were used, in which mice were either challenged intragastrically with *E. coli* O157:H7 or injected intraperitoneally with Shiga toxin 2. An in vitro model of thrombotic microangiopathy was developed in which Shiga toxin 2- and *E. coli* O157 lipopolysaccharide-stimulated human blood cells combined with ADAMTS13-deficient plasma were perfused over glomerular endothelial cells. Two group statistical comparisons were performed using the Mann–Whitney test, multiple groups were compared using the Kruskal–Wallis test followed by Dunn’s procedure, the Wilcoxon signed rank test was used for paired data, or linear regression for continuous variables.

**Results:**

HUS patients had excessively high plasma arginase 1 levels and activity (conversion of L-arginine to urea and L-ornithine) during the acute phase, compared to remission and controls. Arginase 1 levels correlated with lactate dehydrogenase activity, indicating hemolysis, as well as the need for dialysis treatment. Patients also exhibited high levels of plasma alpha-1-microglobulin, a heme scavenger. Both mouse models exhibited significantly elevated plasma arginase 1 levels and activity. Plasma arginase 1 levels correlated with lactate dehydrogenase activity, alpha-1-microglobulin and urea levels, the latter indicative of kidney dysfunction. In the in vitro model of thrombotic microangiopathy, bioactive arginase 1 was released and levels correlated to the degree of hemolysis.

**Conclusions:**

Elevated red blood cell-derived arginase was demonstrated in HUS patients and in relevant in vivo and in vitro models. The excessively high arginase levels correlated to the degree of hemolysis and kidney dysfunction. Thus, arginase inhibition should be investigated in HUS.

**Supplementary Information:**

The online version contains supplementary material available at 10.1186/s12967-023-04824-x.

## Background

This study addressed the release of active arginase in thrombotic microangiopathy (TMA). TMA is a pathological lesion associated with nonimmune hemolytic anemia with fragmented red blood cells, consumptive thrombocytopenia and organ damage. TMA is characterized by extensive endothelial injury and detachment as well as microthrombi in capillaries, arterioles and small arteries, leading to occlusion and secondary ischemia [1, 2]. TMA is subdivided based on etiology into major forms including hemolytic uremic syndrome (HUS) caused by Shiga toxin-producing enterohemorrhagic *Escherichia coli* (EHEC), or atypical HUS, associated with complement overactivation, as well as thrombotic thrombocytopenic purpura (TTP) [2], associated with deficiency or dysfunction of ADAMTS13 (A Disintegrin And Metalloproteinase with a ThromboSpondin 1 motif, member 13) [3]. All forms of TMA are associated with hemolytic anemia.

Elevated circulating arginase has been associated with a range of pathological states, notably cardiovascular, kidney, neurological and hematological diseases [4]. Arginase 1 is released during hemolysis and elevated levels have been described in hemolytic diseases, such as sickle cell disease and thalassemia. Furthermore, it has been shown to be associated with the severity of disease [5] as reflected by cardiovascular dysfunction and pulmonary hypertension [6, 7]. Similarly, patients with paroxysmal nocturnal hematuria, a hemolytic disorder caused by complement activation, exhibit high plasma arginase 1 levels associated with hemolysis [8].

There are two isoforms of arginase, arginase 1 is localized in the cytosol and arginase 2 in mitochondriae [9]. Arginase 1 is expressed predominantly in red blood cells (RBCs), hepatocytes, and macrophages, and arginase 2 in the kidney, brain, retina and other tissues [4]. In addition to its release during cell lysis [6], exocytosis of arginase 1-containing granules from neutrophils [10] or arginase-positive platelet-derived extracellular vesicles [11] has been demonstrated.

Arginase cleaves L-arginine. Arginine is also a substrate of nitric oxide synthase (NOS) and, as such, plays an important role in the balance between two catalytic processes [12]. In the one process arginase participates in the final phase of the urea cycle cleaving L-arginine to urea and L-ornithine. This step is of crucial physiological importance in detoxifying ammonia as well as generating L-ornithine, which is further enzymatically processed to proline and polyamines, ultimately leading to collagen formation, and playing an important role in cell proliferation and tissue repair [13]. In the other process L-arginine is processed by NOS to nitric oxide (NO) and citrulline [14]. In endothelial cells this is catalyzed by endothelial NOS (eNOS). Excess arginase will deplete L-arginine, and thereby NO bioavailability, and lead to endothelial dysfunction [15]. NO depletion is associated with oxidative stress, as a result of superoxide generation by eNOS, and contributes to vasoconstriction and thrombosis [14].

As TMA is a hemolytic condition with marked endothelial cell injury and thrombosis, this study investigated arginase levels and activity in two cohorts of patients with EHEC-associated HUS, as well as two murine models of HUS, induced by EHEC infection or Shiga toxin injection. Furthermore, an in vitro model of TMA using EHEC virulence factors perfused with blood cells, combined with TTP plasma, over endothelial cells was utilized to study the correlation between hemolysis and arginase 1 release in this condition.

## Methods

### Subjects

Samples from two cohorts of pediatric patients with post-enteropathic HUS, as well as two control cohorts, were used in this study. Table 1 summarizes all participating patients and controls. The first cohort consisted of 24 children (0–17 years old) with EHEC-associated HUS treated at the Department of Pediatrics, Skåne University Hospital in Lund, Sweden. All patients treated between the years 1995–2021 from whom sufficient blood samples were available were included. Fourteen patients were reported previously [16–18]. All had prodromal diarrhea. EHEC infection was demonstrated by a positive stool polymerase chain reaction test for virulence factor genes *stx1*, *stx2*, *eaeA* or *uidA* as per hospital routines. HUS was defined as hemolytic anemia (hemoglobin < 100 g/L), thrombocytopenia (platelet count < 140 × 10^9^/L) and acute kidney injury (a greater than 50% increase in plasma creatinine from baseline, or urine output less than 0.5 ml/kg/hour for at least six hours). Table 2 presents the characteristics of this cohort.

**Table 1:** Patients and controls included in this study

Subjects	n	Age years, median (range)	M/F	Sample	Analysis
HUS cohort (Lund)	24	2.5 (0.25–14)	16/8	Plasma	Arginase 1 levels and activity, A1M
Controls to HUS cohort (Lund)	8	10 (1–15)	5/3	Plasma	Arginase 1 levels and activity, A1M
HUS cohort (Philadelphia)	23	3 (0.5–14)	11/12	Serum	Arginase 1 levels
Controls to HUS cohort (Philadelphia)	26	8.5 (6–15)	16/10	Serum	Arginase 1 levels
TTP patients	3	9 (7–21)	2/1	Plasma	In vitro TMA model
Healthy controls	4	Adults	3/1	Whole blood, Plasma	In vitro TMA model, RBC lysate-Arginase 1 level

*A1M* Alpha-1-microglobulin, *HUS* Hemolytic uremic syndrome, *M/F* male/female, *RBC* red blood cell, *TTP* Thrombotic thrombocytopenic purpura, *TMA* thrombotic microangiopathy

**Table 2:** Characteristics of the first hemolytic uremic syndrome cohort (Lund)

Patient	Sex	Age yrs	Bloody diarrhea	Dialysis	*E. coli* serotype	Toxin	Hb^a^ g/L	Plt^a^ × 10^9^/L	LDH^b^ µkat/L
1 ^c^	M	3	+	+	O157	Stx2	72	44	10
2 ^c^	M	1	−	−	O26	Stx2	58	27	87
3 ^c^	M	7	−	+	O157	Stx2	56	18	135
4 ^d^	F	1	+	+	NT	Stx2	63	56	97
5 ^e^	M	1	+	+	O153	Stx2	60	49	40
6	M	14	+	−	O157	Stx2	79	24	25
7	M	2	+	−	ND	Stx2	60	46	23
8	M	3 mo	+	−	ND	Stx2	60	13	36
9 ^c^	M	1	+	−	O145	Stx2	63	31	65
10 ^d^	F	3	+	+	O157	Stx2	77	42	145
11 ^d^	F	2	+	+	O145	Stx2	92	35	−
12 ^d^	M	1	+	−	O157	Stx2	90	16	76
13 ^c^	M	10	−	+	O157	Stx2	49	35	65
14 ^f^	F	1	−	-	ND	ND	73	94	16
15 ^c^	F	2	−	−	O157	Stx2	43	10	111
16 ^e^	F	6	+	+	Non-O157	Stx2	60	10	79
17 ^e^	M	6	+	+	O157	Stx1, Stx2	65	20	44
18 ^c^	M	10	+	+	O145	Stx2	41	30	102
19	M	9	+	+	ND	Stx2	75	14	41
20	M	1	+	+	ND	Stx2	69	9	30
21	F	1	+	+	ND	Stx1, Stx2	74	15	52
22	F	3	+	−	ND	Stx2	79	78	12
23	M	4	+	+	ND	Stx2	70	34	28
24	M	4	+	+	ND	Stx1, Stx2	67	28	32

*M* male, *F* female, *Hb* hemoglobin, *LDH* lactate dehydrogenase activity, *ND* not determined, *NT* non-typeable, *Plt* Platelet count, *Stx* Shiga toxin

^a^ Lowest during the acute disease phase

^b^ Highest during the acute disease phase

^c^ This patient was previously described (17)

^d^ This patient was previously described (16)

^e^ This patient was previously described (18)

^f^ This patient had negative EHEC test results in stool; two household members had positive polymerase chain reaction test result for *stx*

Control plasma samples to the first HUS cohort were available from pediatric patients without HUS (n = 8), with suspected or confirmed kidney disease who underwent iohexol clearance testing at the Department of Pediatrics, Skåne University Hospital. These patients were diagnosed with IgA nephropathy, IgA vasculitis, congenital abnormalities of the kidneys and urinary tract, and chronic kidney disease. Their median estimated glomerular filtration rate was 98 ml/min/1.73m^2^ (interquartile range 71–113 mL/min/1.73m^2^).

The second HUS cohort comprised 23 pediatric patients treated at the Children’s Hospital of Philadelphia, PA, and samples were available after a previous study [19]. These samples were anonymized and no data other than sex, age and day of sampling were available.

Control serum samples to the second HUS cohort were from pediatric patients seen at the outpatient clinic of the Department of Pediatrics, Skåne University Hospital (n = 26). These patients were investigated or treated for conditions other than kidney disease and were previously described [20]. These samples were anonymized.

Samples from three patients with congenital TTP, deficient in ADAMTS13, were used in certain experiments. These patients have been previously described [21, 22]. Control samples were available from healthy adult donors without ongoing medications (n = 4) (Table 1).

### Clinical laboratory parameters

Hemoglobin levels and platelet counts were analyzed on an automated cell counter, and serum lactate dehydrogenase (LDH) activity measured using a colorimetric method, at the hospital’s clinical laboratory as per hospital routines.

### Blood samples

Blood samples were obtained on the day of hospitalization or within two days for patients in both HUS cohorts. Samples were taken before the start of peritoneal dialysis. Remission samples were available for 12/24 patients in the first (Lund) HUS cohort and collected at a median of three weeks (range 1 week–4 years) after the initial hospitalization. In the first HUS cohort, as well as the eight pediatric kidney disease patients used as controls to the first HUS cohort, venous or capillary blood samples were collected in sodium citrate vacutainer or microtainer tubes (Becton Dickinson, Franklin Lakes, NJ). Platelet-poor plasma was obtained after serial centrifugation, first at 1500 × g followed by 10,000 × g, after which the supernatant was frozen and stored at − 80 °C until used. In the second (Philadelphia) HUS cohort, and in the pediatric patients without kidney disease used as their controls, serum samples were collected and stored at − 80 °C until used.

Blood samples from TTP patients were collected in sodium citrate vacutainer tubes. Platelet-poor plasma was obtained after serial centrifugation as described above and stored at − 80 °C until use for in vitro experiments.

Samples from healthy adult volunteers were used in certain experiments. Venous whole blood was drawn using a BD Vacutainer 23G blood collection set (Becton Dickinson) into S-Monovette hirudin tubes (Sarstedt, Nümbrecht, Germany) or sodium citrate vacutainer tubes. The tubes were inverted thoroughly and kept at rt for up to 30 min before use in in vitro experiments or for preparation of RBC lysates (Table 1). Alternatively, platelet-poor plasma was prepared by serial centrifugation as described above and stored at − 80 °C until used.

### Arginase 1 levels, arginase activity, and alpha-1-microglobulin levels

Arginase 1 concentration in human plasma and serum and cell lysates was measured using the Arginase Liver Type Human ELISA kit (Biovendor, Brno, Czech Republic) according to the manufacturer’s instructions. Levels in serum are higher than in plasma. Arginase activity, assayed as the conversion of L-arginine to L-ornithine and urea, was measured in plasma samples and cell culture supernatants using the Quantichrom Arginase Assay Kit (BioAssay Systems, Hayward, CA) according to the manufacturer’s instructions. The assay measures urea formed in the sample per unit time. Plasma samples were filtered using Millipore Amicon Ultra 0.5 ml 10 kDa centrifugal filters (Merck, Darmstadt, Germany) to remove urea before assaying. Alpha-1-microglobulin (A1M) was measured in human plasma using the Alpha 1-Microglobulin ELISA Kit (Aviva Systems Biology, San Diego, CA). In all the assays above, detection was carried out using a Glomax Discovery System (Promega, Madison, WI).

### Mouse model of *E. coli* O157:H7 infection

BALB/c mice were bred in the animal facilities of the Centre for Comparative Medicine, Medical Faculty, Lund University. Female mice aged 8–12 weeks were used (n = 21). The choice of BALB/c mice over other murine strains, such as C57BL/6, was based on previous results showing a more pronounced disease phenotype [23–25]. A streptomycin-resistant derivative of the Shiga toxin 2 (Stx2)-producing enterohemorrhagic *E. coli* O157:H7 strain 86–24 was previously described [25, 26] and was used in the present study. The EHEC infection protocol was performed as previously detailed [23, 24, 27]. Briefly, prior to inoculation, mice were exposed to streptomycin in drinking water (5 g/L) for 24 h and fasted for food for 16 h. Under isoflurane anesthesia (Forene, Abbott, Wiesbaden, Germany) mice were inoculated with 100 µL of bacterial suspension (10^9^ CFU/mL in 20% sucrose and 10% NaHCO_3_) by oral gavage using a soft polyethylene catheter (Clay Adams, Parsippany, NJ). After inoculation, unlimited access to food was reintroduced. Streptomycin treatment was either discontinued on day 2 or continued throughout the experiment. Animals were weighed daily and monitored at least twice daily, for up to nine days, for signs of disease such as ruffled fur, abnormal body positioning or decreased activity. Display of any sign of disease or weight loss ≥ 20% was considered indication for euthanasia by cervical dislocation and occurred on average on day 6. Unaffected mice were sacrificed on day 6–9. Tail snip blood samples were collected in microvette tubes with EDTA anticoagulation (Sarstedt) for blood cell counts. Blood samples for plasma preparation were collected before euthanasia under isoflurane anesthesia by heart puncture and anticoagulated with sodium citrate (Sigma-Aldrich, St Louis, MO). Plasma was prepared by serial centrifugation at 1500 × g for 15 min and 13 000 × g for 3 min. The plasma was stored at − 80 °C until analyzed for urea, arginase 1 levels and activity, LDH activity, and A1M. Kidneys were collected and fixed in 4% paraformaldehyde (Histolab, Gothenburg, Sweden).

### Platelet and neutrophil counts, urea, arginase 1 levels, arginase activity, LDH activity, A1M in murine samples

Platelet and neutrophil counts were performed in EDTA-anticoagulated whole blood on a Sysmex XN-350 (Sysmex Europe, Norderstedt, Germany) automated cell counter and adjusted for weight change. Urea concentration was measured in mouse plasma using the Quantichrom Urea Assay Kit (BioAssay Systems). Arginase 1 concentration in mouse plasma was measured using a Mouse Arginase 1 ELISA Kit (Abcam, Amsterdam, Netherlands). Arginase activity was measured using the same assay as in human plasma. LDH activity was measured in mouse plasma using Lactate Dehydrogenase Activity Assay Kit (Sigma-Aldrich). Plasma A1M levels were measured using the Mouse alpha-1-microglobulin ELISA Kit (Novus Biologicals, Centennial, CO). The Glomax Discovery System was used for detection.

### Histopathological analysis

Kidneys collected from mice upon sacrifice were fixed in 4% paraformaldehyde (Histolab), embedded in paraffin and sectioned (3 µm). Sections were stained with hematoxylin and eosin, coded, and blinded to the investigators. Images were captured using a Nikon Eclipse Ti-E microscope with Nikon color camera using NIS Elements AR software v.5.11.01 (Nikon Instruments Inc., Tokyo, Japan). Images of entire transversal kidney sections were analyzed by two investigators separately and results were subsequently averaged. For each animal treated with *E. coli* O157:H7 (n = 9) or phosphate-buffered saline (PBS, n = 4), one entire kidney section was analyzed. Pathology was scored as 0: absent, 1: mild, 2: moderate, or 3: severe, and scored for tubular cell vacuolization, inflammatory cell infiltrates, tubular epithelial desquamation, interstitial edema, blood cells in the tubular lumen, congestion of glomerular capillaries and arterioles as well as mesangial proliferation.

### Detection of kidney alpha 1 microglobulin by immunohistochemistry

Unstained entire kidney sections were obtained as above, deparaffinized, and fluorescently labeled using polyclonal rabbit anti-mouse A1M antibody (developed in-house) [28] and Alexa 488-coupled goat anti-rabbit secondary antibody (Invitrogen, Waltham, MA) and mounted with Prolong Diamond antifade mountant with DAPI (4',6-diamidino-2-phenylindole; Invitrogen). Images were obtained from one entire kidney section per mouse treated with *E. coli* O157:H7 (n = 9) or PBS (n = 4). Images were captured using a Nikon Eclipse Ti-E microscope at 20 × magnification with a Hamamatsu flash camera and NIS Elements AR software (Nikon) and merged using Fiji software [29]. Stitched images of one entire kidney section per animal were qualitatively evaluated in a blinded manner for staining.

### Mouse model of Shiga toxin injection

Male (n = 6) and female BALB/c (n = 13) mice aged 8–12 weeks were used. Shiga toxin 2 (Phoenix Lab, Tufts Medical Center, Boston, MA) was diluted in PBS and injected intraperitoneally at 142 ng/kg weight as previously described [30] (n = 12, 3 male, 9 female). The endotoxin content of Stx2 was tested using the LAL Chromogenic Endotoxin Quantification kit (Pierce Biotechnology, Rockford, IL) and found to be < 0.2 ng per 1 µg Stx2. Control mice were injected with the same volume of PBS (n = 7, 3 male, 4 female). Mice were monitored daily and euthanized upon exhibiting clinical signs of disease, which occurred on average 4 days after injection. Blood samples for blood cell counts and plasma were prepared as above. The plasma was stored at − 80 °C until analysis for urea and arginase 1 levels and arginase activity.

### In vitro microfluidic model of thrombotic microangiopathy

Primary human glomerular microvascular endothelial cells (PGEC; Cell Systems, Kirkland, WA) were cultured in Endothelial cell growth basal medium-2, supplemented with EGM-2 MV Microvascular Endothelial SingleQuots Kit (both from Lonza, Walkersville, MD) including 5% fetal bovine serum, and 1 × penicillin/streptomycin (Fisher Scientific, Loughborough, UK). The cells were grown at 37 °C in 5% CO_2_ and used between passages 7–11. Before the start of experiments cells were detached using 1 × TrypLE (Life Technologies, Grand Island, NY), washed once, and resuspended in the same medium without serum.

Vena8 Endothelial + biochip capillaries (Cellix, Dublin, Ireland) precoated with 100 µg/mL bovine fibronectin (Sigma-Aldrich) were seeded with the PGEC suspension (0.2 million cells/10 µL/capillary) and incubated for 2.5 h at 37 °C in 5% CO_2_. Fresh serum-free medium, with or without Stx2 200 ng/mL and *E. coli* O157 lipopolysaccharide 1 µg/mL (O157LPS; Nacalai Tesque Inc, Kyoto, Japan), together or separately, was added to the capillary inlet and outlet wells after the first 30 min.

Hirudin-anticoagulated whole blood from healthy volunteers (blood group O) was incubated with or without Stx2 200 ng/mL and O157LPS 1 µg/mL, together or separately, for 90 min at 37 °C. Blood cells were pelleted by centrifugation at 1500 × g for 10 min, the plasma was removed, and cells were resuspended in ADAMTS13-deficient TTP plasma diluted 1:2 in isotonic Krebs–Ringer solution (Thermo Fisher, Kandel, Germany). The blood cell suspension was used immediately for perfusion experiments.

Following PGEC incubation with Stx2 and O157LPS, the biochip outlets were connected to a Mirus Evo pump controlled by VenaFlux64 software (both from Cellix) via 3.5 mL plastic collection tubes (Sarstedt). Blood cell suspensions were perfused through the biochip capillaries into the collection tubes at a speed of 55 µL/min and an average estimated shear stress of 2 Pascal. This shear stress was chosen to mimic renal glomerular shear stress [31] and was calculated using the formula $\tau=\frac{6Q\mu}{wh^2}$ [32], in which τ is the shear stress, Q is the volumetric flow rate, µ is the estimated blood viscosity (0.0045 Pa·s) [33], and w and h the width and height of the capillary. Perfused samples were collected and centrifuged at 2000 × g for 10 min. The supernatant was then further centrifuged at 10,000 × g for 10 min. The perfusion experiments were performed at 37 °C and centrifugations were performed at rt. Optical density (OD) at 405 nm was assessed as a measure of hemolysis on a Glomax Discovery System. The samples were kept at − 20 °C until analyzed for arginase 1 and arginase activity.

In a separate set of experiments, healthy donor whole blood was incubated with or without Stx2 200 ng/mL and O157LPS 1 µg/mL for 90 min at 37 °C and then immediately perfused through microcapillaries coated with PGEC preincubated with Stx2 and O157LPS, as above. Plasma was prepared from perfused samples and hemolysis was assessed as above.

### PGEC and RBC lysate preparation

PGEC were cultured to confluence, washed twice with cold PBS (Hyclone, Logan, UT) and scraped in RIPA lysis buffer with protease inhibitor cocktail (Santa Cruz Biotechnology, Dallas, TX). PGEC lysates were freeze-thawed once and centrifuged at 10,000 × g for 10 min at 4 °C. Supernatants were stored at -20 °C until analyzed for levels of arginase 1. Protein concentration was assessed by the Bicinchoninic assay (Sigma-Aldrich).

For determination of intracellular RBC arginase 1, RBC lysates were prepared. Briefly, whole blood from healthy adult volunteers was centrifuged at 150 × g for 15 min at rt. The RBC pellet was then washed four times in large amounts of PBS without calcium and magnesium (Hyclone), followed by centrifugation at 500 × g at rt. After each centrifugation, the supernatant was fully removed together with the supernatant-pellet interface to remove white blood cells. The RBC solution at 150 µL was then incubated with dH_2_0 1350 µL for 20 min with gentle rocking at 4 °C, followed by centrifugation at 2500 × g for 15 min at 4 °C. Protein concentration was determined by the Bicinchoninic assay and supernatants stored at − 20 °C until used for measurement of arginase 1 levels.

### Statistical analysis

Data were assessed for normality and appropriate transformations were performed accordingly. Two-group comparisons of continuous numerical data were carried out using the non-parametric Mann–Whitney U test with effect size expressed as probabilistic index. Multiple group comparisons were performed using the Kruskal–Wallis test followed by Dunn’s multiple comparisons test using Bonferroni adjustment with effect size expressed as Cohen’s r. Paired data were assessed using the non-parametric Wilcoxon signed rank test with effect size expressed as median difference in location. Pairs of in vitro microfluidic perfusion experiments of healthy donor blood cells preincubated with Stx2 and O157LPS or PBS were performed on the same day, using the same donor. These two experimental groups were not considered independent and were thus analyzed as paired data by the Wilcoxon signed rank test. Correlation between continuous variables was assessed using simple linear regression. Data were visualized using Graph Pad Prism version 9.2.0 (Graph Pad Software, San Diego, CA) and statistical analyses were performed using R version 4.2.0 (R Core Team, 2022). A P value ≤ 0.05 was considered significant.

## Results

### Arginase activity was elevated in plasma of patients with EHEC-HUS

**Fig. 1:**
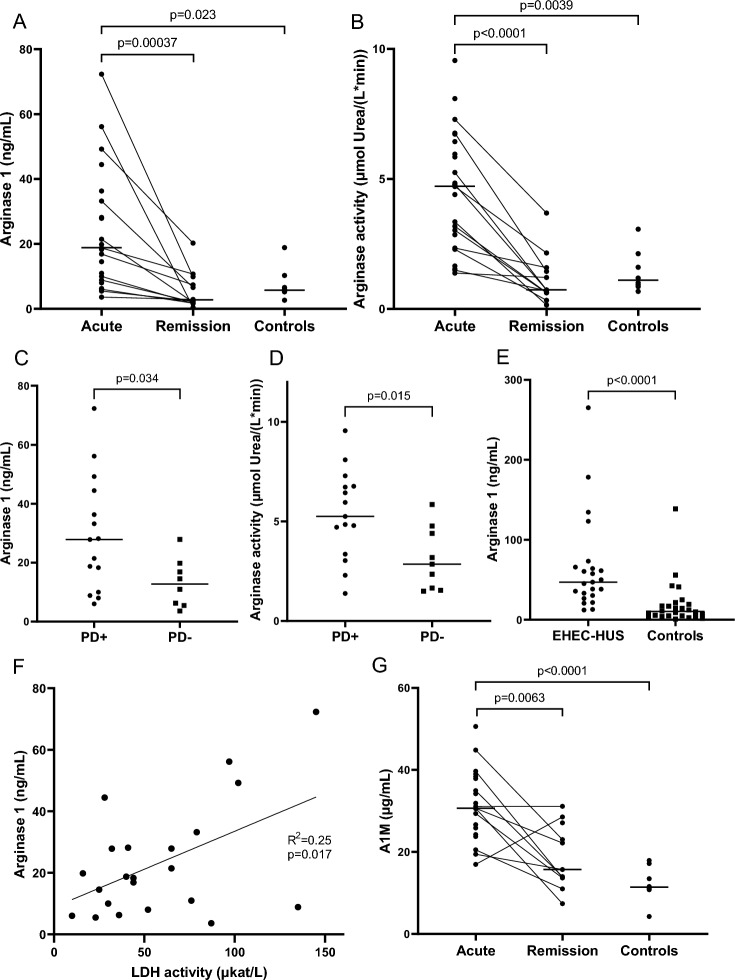
Arginase levels in pediatric patients with EHEC-HUS. Blood samples from pediatric patients with acute enterohemorrhagic *E. coli*-associated hemolytic uremic syndrome (EHEC-HUS) at admission or during remission and from non-HUS pediatric controls were analyzed for arginase 1 levels and activity. **A** Plasma arginase 1 was elevated in acute EHEC-HUS (first cohort, n = 23, median 18.7 ng/mL; one sample not analyzed due to small volume) compared to remission (n = 12, median 2.62 ng/mL) and pediatric controls (n = 8, median 5.73 ng/mL). **B** Arginase activity in the same patients (n = 24) and controls as in A. **C** Plasma arginase 1 in patients with acute EHEC-HUS who required peritoneal dialysis (n = 15, median 27.9 ng/mL) compared to those that did not (n = 8, median 12.7 ng/mL; one sample not analyzed due to small volume). **D** Plasma arginase activity in patients with acute EHEC-HUS who required peritoneal dialysis (n = 15, median 5.25 µmol urea per L and minute) compared with those that did not (n = 9, median 2.85 µmol urea per L and minute). **E** Serum arginase 1 in the Philadelphia cohort of pediatric patients with acute EHEC-HUS (n = 23, median 47.1 ng/mL) and controls (n = 26, median 10.4 ng/mL). **F** Plasma arginase 1 correlated with the hemolysis biomarker lactate dehydrogenase activity in pediatric patients with acute EHEC-HUS (Lund cohort, n = 22; lactate dehydrogenase activity data lacking in one patient and arginase 1 level not analyzed in one patient). **G** Alpha-1-microglobulin plasma levels during acute EHEC-HUS (n = 21, Lund cohort, median 30.7 µg/mL; three samples not analyzed due to small volume), at remission (n = 11, median 15.7 µg/mL) and in pediatric controls (n = 8, median 11.3 µg/mL). Bars denote medians. Comparisons performed using Kruskal–Wallis test followed by Dunn’s multiple comparisons test (panels A, B and G), Mann–Whitney U test (panels C-E), or simple linear regression (panel F). For detailed statistics see Additional File 1. *A1M* Alpha-1-microglobulin, *EHEC-HUS* Enterohemorrhagic *E. coli*-associated hemolytic uremic syndrome, *LDH activity* Lactate dehydrogenase activity, *PD* Peritoneal dialysis

Arginase was measured in blood samples from pediatric EHEC-HUS patients and controls. The first HUS cohort consisted of 24 patients (described in Tables 1 and 2). Plasma arginase 1 (Fig. 1A) was significantly elevated at admission compared with samples taken during remission and pediatric non-HUS controls (n = 8). Arginase activity, measuring the conversion of L-arginine to L-ornithine and urea, was likewise exceedingly high in the acute phase (Fig. 1B). Patients who required peritoneal dialysis during their hospital stay, due to more severe kidney injury, had higher plasma arginase 1 levels (Fig. 1C) and arginase activity (Fig. 1D) at admission.

To confirm the results of the first HUS cohort a second cohort of patients was examined. Serum samples from this cohort of 23 pediatric patients with post-enteropathic HUS displayed significantly increased levels of arginase 1 compared with pediatric control serum samples (n = 26; Fig. 1E). Additional file 1 provides a detailed summary of the statistical tests performed in the same order as the figures.

### Arginase in plasma correlated with hemolysis markers LDH activity and alpha-1-microglobulin

Arginase 1 is abundant in the cytoplasm of RBCs. LDH activity was used as a biomarker for the degree of hemolysis and correlated with plasma arginase 1 concentration in patients with acute EHEC-HUS (first cohort; Fig. 1F) suggesting that circulating arginase 1 originated from fragmented RBC.

As an additional biomarker related to hemolysis, A1M was measured. A1M is a heme and radical oxygen species scavenger which is upregulated in response to oxidative stress and protects cells and tissues from heme- and oxidative stress-associated injury [34]. Plasma A1M was elevated in acute EHEC-HUS samples from the same cohort compared with samples taken during remission and pediatric controls (Fig. 1G). One patient with a higher A1M level during remission than at admission was a 1-year-old girl with severe acute kidney injury that required peritoneal dialysis; the remission sample was taken 3 months after disease onset.

### Disease phenotype in *E. coli* O157:H7 infected mice

**Fig. 2:**
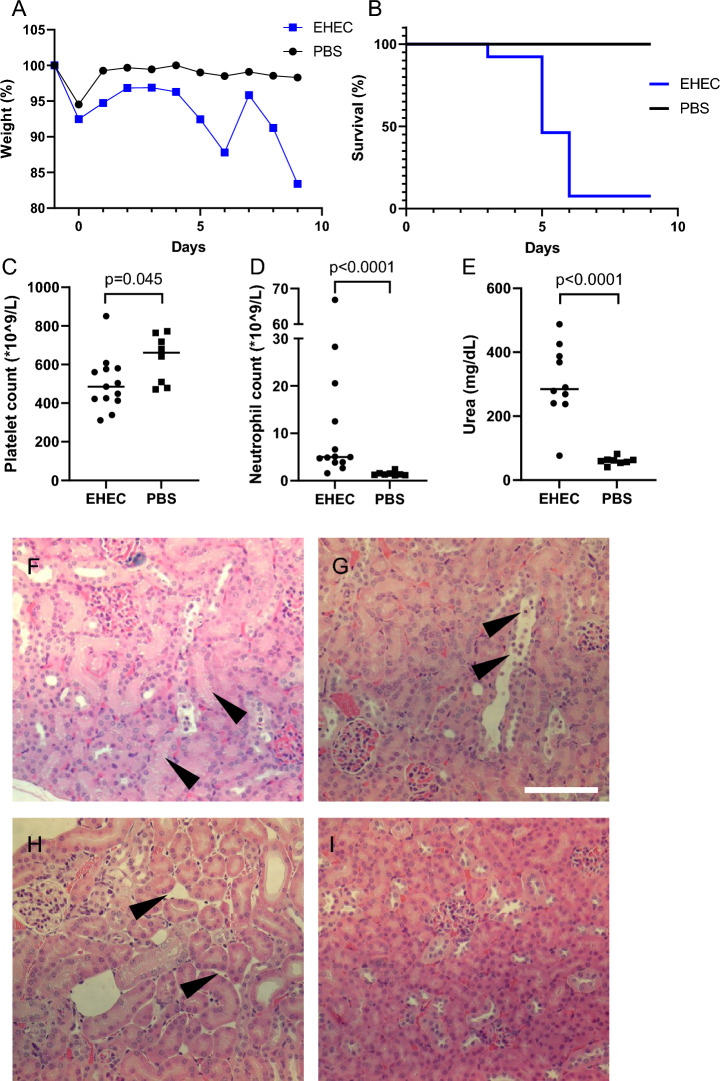
Mouse model of EHEC infection: clinical disease, survival and kidney injury. BALB/C mice were inoculated intragastrically with *E. coli* O157:H7 or PBS vehicle and when signs of disease developed or at end of experiment (days 6–9) blood samples and kidneys were collected. **A** Body weight change in mice inoculated with *E. coli* O157:H7 (n = 13) or vehicle controls (n = 8) presented as medians. **B** Survival plot of the same mice as in A. **C** Platelet counts in mice infected with *E. coli* O157:H7 (n = 13, median 486 × 10^9^/L) and PBS vehicle controls (n = 8, median 662 × 10^9^/L). **D** Neutrophil counts in mice infected with *E. coli* O157:H7 (n = 13; median 5.0 × 10^9^/L) and PBS (n = 8; median 1.3 × 10^9^/L). **E** Plasma urea in mice inoculated with *E. coli* O157:H7 (n = 10; three samples not analyzed, median 285 mg/dL) and PBS controls (n = 8, median 60.7 mg/dL). Bars denote medians. **F** A representative kidney section from a mouse infected with *E. coli* O157:H7. Arrowheads indicate tubular vacuolization. **G** Representative kidney section from a mouse infected with *E. coli* O157:H7. Arrowheads indicate tubular epithelial desquamation. **H** Representative kidney section from a mouse infected with *E. coli* O157:H7. Arrowheads indicate interstitial edema. **I** Representative kidney section from a control mouse showing normal mouse kidney tissue. Scale bar: 100 µm. Comparisons performed using Mann–Whitney U test (panels C-E). *EHEC* Enterohemorrhagic *E. coli*, *PBS* phosphate-buffered saline

BALB/c mice inoculated intragastrically with *E. coli* O157:H7 developed signs of disease on average on day 5–6. Sick mice exhibited weight loss (Fig. 2A) and were euthanized when disease developed (Fig. 2B). *E. coli* O157:H7-infected mice displayed decreased platelet counts (Fig. 2C) and elevated neutrophil counts (Fig. 2D) compared with control mice. *E. coli* O157:H7-infected mice also displayed elevated plasma urea compared with control mice (Fig. 2E). Histopathological assessment of kidney sections showed increased tubular cell vacuolization and desquamation, as well as interstitial edema in mice inoculated with *E. coli* O157:H7 (n = 9, four animals were not analyzed; Fig. 2F–H), indicating acute kidney injury [25, 35] compared to control mice (n = 4, four animals were not analyzed, Fig. 2I). There was mild congestion of glomerular capillaries and arterioles in both groups. Mesangial proliferation, blood cells in tubular lumens and inflammatory cell infiltrates were not seen. Quantification of the histopathological analysis is presented in Table 3. Our group has previously demonstrated findings indicating hemolytic anemia in *E. coli* O157:H7-infected mice that developed kidney injury [25, 36]. Mice inoculated with *E. coli* O157:H7 develop hemolysis with fragmentation of red blood cells [36] and decreased red blood cell counts as well as platelet counts [25]. In the present study, blood was collected after clinical signs of disease developed, and the severe weight loss, due to dehydration, precluded accurate interpretation of red blood cell counts (which increase during hemoconcentration) and are therefore not shown. The degree of hemolysis was assessed by levels of LDH and A1M, described in the following section.

**Table 3:** Kidney histopathology in *E. coli* O157:H7-infected mice

Histopathological finding	*E. coli* O157:H7-infected (n = 9)	Controls (n = 4)
Tubular vacuolization	2 (0–2) ^a^	1 (0–2)
Tubular epithelial desquamation	1.5 (0–3)	0 (0–0.5)
Interstitial edema	2 (0–2)	0.5 (0–1)

^a^ Scores represent median (range). For each mouse one entire kidney section was analyzed. Lesions were scored blindly as 0: absent, 1: mild, 2: moderate, or 3: severe

### Elevated plasma arginase in *E. coli* O157:H7-infected mice

**Fig. 3:**
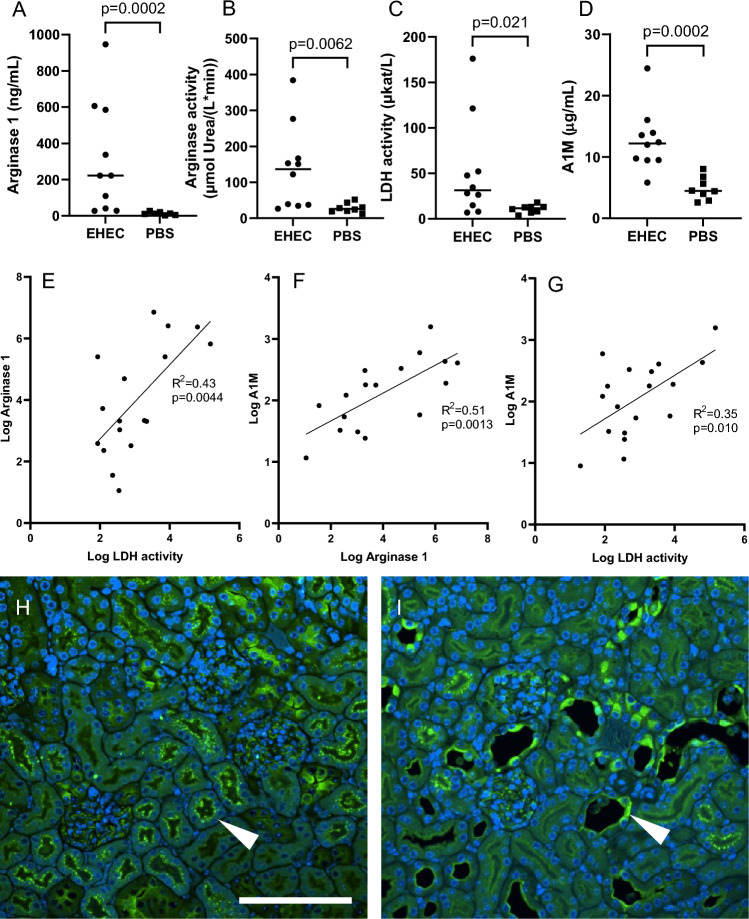
Arginase and hemolysis markers in EHEC-infected mice. **A** Plasma arginase 1 levels in mice inoculated with *E. coli* O157:H7 (n = 10, three samples not analyzed; median 222 ng/mL) and PBS vehicle controls (n = 7; one sample not analyzed; median 12.4 ng/mL). **B** Plasma arginase activity in mice inoculated with *E. coli* O157:H7 (n = 10, three samples not analyzed, median 136 µmol urea per L and minute) and PBS controls (n = 8, median 26.1 µmol urea per L and minute). **C** Plasma lactate dehydrogenase activity in mice inoculated with *E. coli* O157:H7 (n = 10, three samples not analyzed, median 31.4 µkat/L) and PBS controls (n = 8, median 11.7 µkat/L). **D** Alpha-1-microglobulin levels in plasma of mice inoculated with *E. coli* O157:H7 (n = 10, three samples not analyzed; median 12.2 µg/mL) and PBS controls (n = 8; median 4.48 µg/mL). **E** Correlation between arginase 1 levels and lactate dehydrogenase activity in plasma from mice infected with *E. coli* O157:H7 or vehicle controls (n = 17). **F** Correlation between alpha-1-microglobulin and arginase 1 levels in plasma from mice infected with *E. coli* O157:H7 or vehicle controls (n = 17). **G** Plasma alpha-1-microglobulin levels correlated with plasma lactate dehydrogenase activity in mice infected with *E. coli* O157:H7 or vehicle controls (n = 18). **H** Kidney section from a control mouse labeled with alpha-1-microglobulin antibody (green) and DAPI nuclear staining (blue). Arrowhead points to granular pattern of distribution on the luminal side of tubular cells (arrowhead). **I** Kidney section from a mouse infected with *E. coli* O157:H7, showing deposition at the luminal surface and stronger cytoplasmic staining of tubular cells (arrowhead). Scale bar: 100 µm. Comparisons performed using Mann–Whitney U test (panels A-D) or simple linear regression (panels E–G). Bars in panels A-D denote medians. *A1M* Alpha-1-microglobulin, *DAPI* 4',6-diamidino-2-phenylindole, *EHEC* Enterohemorrhagic *E. coli*, *LDH activity* Lactate dehydrogenase activity, *PBS* phosphate-buffered saline

*E. coli* O157:H7-infected mice displayed elevated plasma arginase 1 compared with control mice (Fig. 3A). Likewise, the mice exhibited increased plasma arginase activity, LDH activity, and A1M (Fig. 3B–D, respectively), suggesting hemolysis in these mice. A significant positive correlation was found between plasma arginase 1 and LDH activity (Fig. 3E), between plasma arginase 1 and A1M (Fig. 3F), and between plasma A1M and LDH activity (Fig. 3G). Furthermore, both plasma arginase 1 and arginase activity correlated with urea (Additional File 2, panels A-B). Taken together, these results show increased plasma arginase associated with increased hemolysis and decreased kidney function in this mouse model of HUS-like disease.

To further explore the effects of hemolysis, the presence of heme scavenger A1M in kidney tissue was assessed by immunofluorescent microscopy. In control mice (n = 4, four animals not analyzed), A1M granular staining was at the luminal side of cortical tubuli (Fig. 3H), consistent with physiological filtration at the glomerulus and tubular reabsorption [37]. In 7/9 mice inoculated with *E. coli* O157:H7 (four animals not analyzed), there was marked cytoplasmic staining of tubular cells (Fig. 3I). In the remaining two examined mice inoculated with *E. coli* O157:H7, the staining pattern was similar to that of control mice. Control sections labeled with only secondary antibody displayed no staining.

### Elevated plasma arginase activity in Stx2-injected mice

**Fig. 4:**
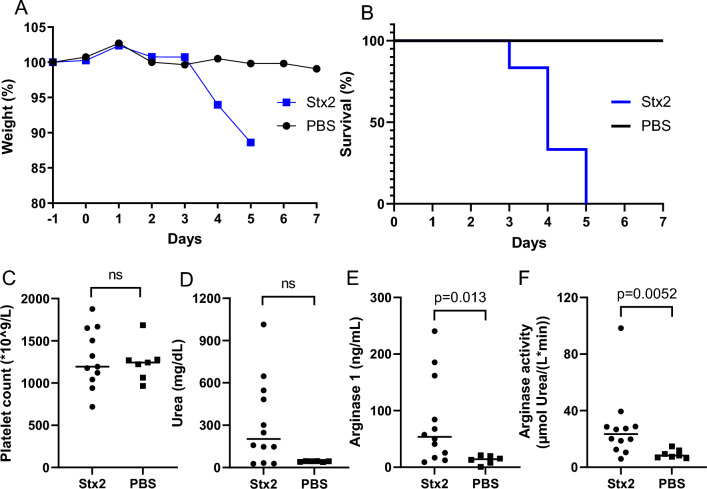
Mouse model of Shiga toxin 2 injection: clinical disease, kidney injury and plasma arginase. **A** Body weight change in mice injected with Shiga toxin 2 (n = 12) or PBS vehicle (n = 7). **B** Survival of mice injected with Shiga toxin 2 (n = 12) or PBS vehicle (n = 7) during the 7-day long experiment. **C** Platelet counts in mice injected with Shiga toxin 2 (n = 11, one animal not analyzed, median 1193 × 10^9^/L) or PBS vehicle (n = 7, median 1243 × 10^9^/L). **D** Plasma urea in mice injected with Shiga toxin 2 (n = 12, median 203 mg/dL) and PBS controls (n = 6; one sample not analyzed, median 44.5 mg/dL). **E** Plasma arginase 1 levels in mice injected with Shiga toxin 2 (n = 12, median 53.7 ng/mL) and control mice (n = 6; one sample not analyzed, median 14.3 ng/mL). **F** Plasma arginase activity in mice injected with Shiga toxin 2 (n = 12, median 23.5 µmol urea per L and minute) and control mice (n = 7, median 8.27 µmol urea per L and minute). Comparisons performed using Mann–Whitney U test (panels D-F). Bars in panels C-F denote medians. *ns* non-significant, *PBS* phosphate-buffered saline, *Stx2* Shiga toxin 2

BALB/C mice were injected intraperitoneally with Stx2 or PBS vehicle, monitored daily, and sacrificed at the first occurrence of disease signs, on average day 4, or at the end of the experiment on day 7 (Fig. 4). Control mice did not exhibit signs of illness and were sacrificed by cervical dislocation on days 5–7 after injection. Mice injected with Stx2 exhibited decreased body weight (Fig. 4A) and survival (Fig. 4B). Platelet counts did not differ between the two groups (Fig. 4C). Neutrophil counts were not available from these mice. Most of the Stx2-injected mice displayed increased plasma urea (Fig. 4D), although the difference compared with control mice was not statistically significant. Stx2-injected mice exhibited significantly increased plasma arginase 1 (Fig. 4E) and arginase activity (Fig. 4F) compared with control mice. Arginase 1 level and arginase activity correlated with plasma urea (Additional File 2, panels C-D), suggesting a link between elevated arginase levels and activity and kidney function. Male and female mice displayed similar responses to the exposure.

### Stx2 and O157LPS induced arginase 1 release from blood cells in an in vitro model of thrombotic microangiopathy

**Fig. 5:**
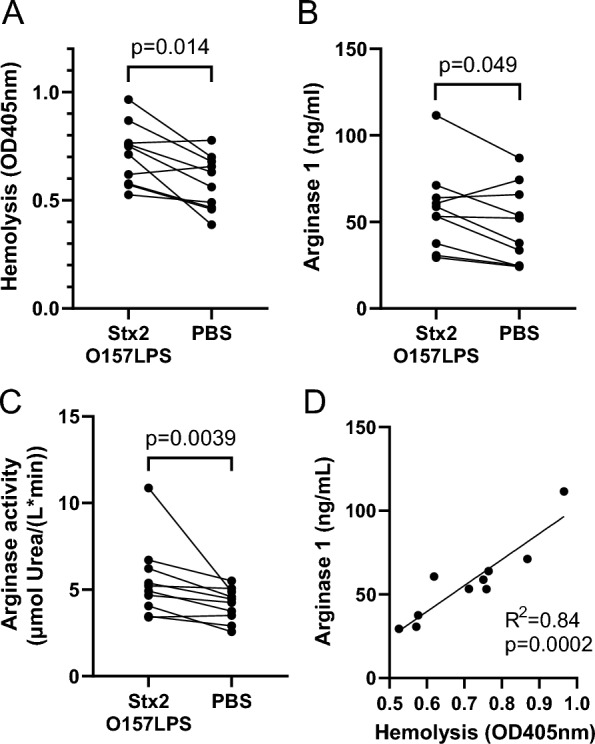
Hemolysis and arginase in an in vitro model of thrombotic microangiopathy. Blood cells that were preincubated with Shiga toxin 2 and lipopolysaccharide from *E. coli* O157:H7 or PBS and suspended in ADAMTS13-deficient plasma were perfused over glomerular endothelial cells. **A** Hemolysis (OD405 nm) in samples preincubated with Shiga toxin 2 and O157LPS (n = 10) or PBS (n = 10). **B** Arginase 1 levels in samples preincubated with Shiga toxin 2 and O157LPS compared with PBS control samples **C** Arginase activity in samples preincubated with Shiga toxin 2 and O157LPS compared with PBS control samples **D** Correlation between arginase 1 levels and hemolysis in samples preincubated with Shiga toxin 2 and O157LPS (n = 10). Comparisons were performed using Wilcoxon signed rank test (panels A-C) or simple linear regression (panel D). *ADAMTS13* A Disintegrin and Metalloproteinase with a ThromboSpondin type 1 motif, member 13, *O157LPS* Lipopolysaccharide from *E. coli* O157:H7, *PBS* phosphate-buffered saline, *Stx2* Shiga toxin 2

A microfluidic system was used to mimic capillary blood flow in the kidney, and blood cells were suspended in ADAMTS13-deficient plasma from patients with congenital TTP to simulate platelet deposition [38] and reflect the prothrombotic microenvironment of glomeruli during TMA. In TTP, platelet adhesiveness is increased [21] due to ADAMTS13 deficiency or dysfunction [3] and the inability to break down ultra-large von Willebrand factor multimers. Blood cell suspensions were perfused through glomerular endothelial cell-coated microcapillaries in an experimental setting investigating if hemolysis and the release of arginase 1 from cells are induced. Samples preincubated with Stx2 and O157LPS exhibited higher levels of hemolysis (Fig. 5A) than PBS control samples and this was accompanied by increased extracellular arginase 1 concentration (Fig. 5B) and arginase activity (Fig. 5C). Arginase 1 concentration correlated with the degree of hemolysis (Fig. 5D). Preincubation with Stx2 or O157LPS alone generated moderate increases in hemolysis (Additional File 3, panel A) and arginase activity (Additional File 3, panel B). To control for the origin of arginase 1, RBC and PGEC lysates were assayed separately, the latter contained undetectable amounts of arginase 1 (Additional File 3, panel C). In a separate set of experiments, healthy donor hirudin-anticoagulated whole blood was pre-treated with Stx2 and O157LPS and perfused over PGEC as above and hemolysis was not observed (Additional File 3, panel D). Likewise, arginase activity was not increased (Additional File 3, panel E).

## Discussion

This study investigated the release of arginase in TMA, specifically in HUS associated with Shiga toxin-producing *E. coli*. HUS patient samples exhibited increased plasma arginase 1 levels and activity, as detected by the conversion of L-arginine to L-ornithine and urea. Using two established mouse models, gastrointestinal infection with *E. coli* O157:H7 and intraperitoneal injection with Shiga toxin 2, elevated circulating arginase 1 and arginase activity were demonstrated and correlated with biomarkers related to hemolysis, such as LDH and the heme scavenger A1M. Increased arginase activity depletes L-arginine, and, as L-arginine is also a substrate of NOS, this will ultimately lead to decreased NO [12]. The findings presented herein provide evidence that excess arginase 1 is released during HUS in correlation to hemolysis and could thereby have an adverse effect on the endothelium in TMA. The study focused on HUS, a condition characterized by acute kidney injury, and demonstrated that elevated arginase correlated to dialysis treatment in patients, and urea levels in mice, suggesting more severe kidney dysfunction. Thus, elevated arginase, by affecting NO catabolism, could contribute to the development of kidney failure.

TMA lesions exhibit pronounced endothelial cell injury, as well as platelet consumption in microthrombi, in the presence of fragmented RBCs. During infection with Shiga toxin-producing *E. coli* extensive damage to the endothelium [39] and platelet activation [40, 41] are caused by the effects of the toxin itself. Our group has previously shown that Shiga toxin can induce hemolysis and the release of extracellular vesicles from RBCs [42]. Lysed RBCs are an important source of arginase 1, and elevated arginase disrupts endothelial homeostasis, by reducing NO and increasing superoxide, thereby contributing to vasoconstriction and thrombosis [6, 14].

The results suggest that the combined effects of Shiga toxin and arginase would severely damage the endothelium and thus arginase inhibition could have therapeutic potential. Arginase inhibitors have been developed, using derivatives of arginine such as *N*^ω^-hydroxy-L-arginine (NOHA) or boronic amino acid derivates such as 2(*S*)-amino-6-boronohexanoic acid (ABH) and *S*-(2-boronoethyl)-L-cysteine (BEC) [43]. Notably, their use in animal models was not toxic [43]. A phase 1/2 trial of an arginase inhibitor in cancer patients has completed patient recruitment [44] and an arginase 1 peptide vaccine was deemed safe in a phase 1 trial [45]. Based on this, arginase inhibition should be further investigated in HUS.

Arginase 1 is released from lysed RBCs in other hemolytic conditions such as sickle cell disease [6], beta-thalassemia [7], and paroxysmal nocturnal hemoglobinuria [8]. In these conditions elevated arginase correlated with disease severity such as the development of pulmonary hypertension [6, 8], and cardiopulmonary dysfunction [7]. Importantly, the hemolytic conditions described above, as well as non-hemolytic conditions in which arginase activity was found to be elevated, such as rheumatoid arthritis [46] and polycystic ovary syndrome [11], do not exhibit TMA lesions. Therefore, arginase most probably contributes to considerable endothelial dysfunction but the specific disease-associated lesion affecting the renal vasculature is caused by Shiga toxin-mediated injury during infection [18]. Kidney failure, occurring during HUS, may further increase arginase levels as a recent study showed an association between plasma arginase activity and chronic kidney disease in children [47] although others have not demonstrated a correlation between plasma arginase and kidney function [6, 48].

The mouse models used display some, but not all, features of human *E. coli* O157-associated HUS. Glomeruli exhibit fibrinogen deposition [25, 49] and both glomerular and tubular cells show pronounced apoptosis [50]. RBC fragmentation was previously reported in our murine studies of EHEC infection [23, 36] and here we show LDH and A1M levels as markers of hemolysis. Furthermore, decreased platelet counts in *E. coli* O157-infected mice suggest thrombocytopenia and elevated neutrophil counts are associated with severe EHEC-associated HUS [51]. Thus, the mice exhibit cardinal features of HUS, acute kidney injury with elevated urea and pathological lesions, thrombocytopenia and hemolysis.

NO is a potent vasodilatory and antithrombotic agent. Due to elevated arginase, reactive oxygen species will be generated instead of NO [14], and, importantly, have opposing effects (i.e. vasoconstrictive and prothrombotic). In EHEC-HUS, decreased NO production and elevated plasma nitrate were reported and correlated with cell-free hemoglobin [52]. Cell-free hemoglobin has strong avidity for NO, which it will remove [53]. Using a baboon model, injected intravenously with Shiga toxin 1, decreased urinary NO metabolites were demonstrated [54] and suggested to be due to the binding of NO to cell-free hemoglobin. In that study arginase activity was not addressed, however, based on our current investigation, using Shiga toxin 2 injected intraperitoneally in mice, we suggest that increased arginase activity could also contribute to decreased NO bioavailability. Of note, arginine therapy may be beneficial [55] and has been suggested for EHEC-HUS due to its ability to generate NO [56].

Cell-free hemoglobin is nephrotoxic, and its release will contribute to endothelial damage and platelet aggregation [57, 58]. Hemoglobin oxidation in the kidney, from the ferrous Fe^2+^ to the ferric ion Fe^3+^ state, contributes to oxidative stress and acute kidney injury [58]. Hemoglobin, and its metabolite heme, are cleared by hemopexin, haptoglobin and A1M [59, 60], however, the effect of these scavengers is often insufficient during fulminant hemolysis. Interestingly, we could show that A1M was elevated during the acute phase of HUS, both in patients and in *E. coli* O157:H7-infected mice. In healthy renal tubular cells we could demonstrate A1M localization at the luminal side of the cell. However, in the kidneys of infected mice, tubular cells were detaching and, in these cells, A1M exhibited an altered distribution. As these cells are shed into the urine, a decreased protective effect of A1M in renal tissue could be a consequence. Likewise, in a murine ischemia–reperfusion model of acute kidney injury tubular A1M staining was reduced [61] and this was suggested to be due to decreased tubular reabsorption. Hemolysis, as in HUS, induces the release of cell-free hemoglobin while the physiological heme and radical oxygen species scavenger A1M is redistributed within damaged tubular cells, suggesting a weaker protective effect. Cell-free hemoglobin depletes NO and could thereby enhance the arginase-mediated injury.

Using a microfluidic perfusion system to mimic renal glomerular capillary shear stress we demonstrated that Shiga toxin 2 and O157LPS, alone or in combination, induced hemolysis and the release of arginase. The experiments were designed to generate a TMA-like lesion using shear and ADAMTS13-deficient plasma, as this model was previously shown to stimulate deposition of von Willebrand factor-platelet strings on the endothelium under flow [38]. Under these conditions hemolysis was induced and active arginase 1 was released, suggesting that this is the mechanism by which arginase 1 is released from lysed RBCs during HUS. Using healthy donor plasma hemolysis was not induced. Of note, the specific conditions mimicking glomerular capillaries may explain the propensity of the kidney to develop injury during HUS, as RBCs are fragmented within occluded glomerular capillaries which could lead to higher arginase concentrations in the renal cortex.

This study demonstrates that bioactive arginase 1 is released during HUS due to hemolysis. Shiga toxin induces marked endothelial cell injury and platelet activation while arginase 1 contributes to the severity of the microvascular lesion by reducing the bioavailability of NO. Future studies should address the combined effects of Shiga toxin and arginase 1 on the endothelium, the induction of TMA and the biological effects of arginase inhibition.

### Supplementary Information

**Additional file 1. **Statistical comparisons performed in this study (Table).

**Additional file 2. **Correlation of arginase and urea in two mouse models. **A) **Plasma urea correlated with plasma arginase 1 in mice inoculated with *E. coli *O157:H7 or vehicle controls (n=17). **B)** Plasma urea correlated with plasma arginase activity in mice inoculated with *E. coli *O157:H7 or vehicle controls (n=17). **C)** Plasma urea correlated with plasma arginase 1 in mice injected with Shiga toxin 2 or vehicle controls (n=18). **D)** Plasma urea correlated with plasma arginase activity in mice injected with Shiga toxin 2 or vehicle controls (n=18). Comparisons performed using simple linear regression.

**Additional file 3. **Hemolysis and arginase in an *in vitro* model of thrombotic microangiopathy. Blood cells were pre-incubated with Shiga toxin 2 and/or lipopolysaccharide from *E. coli* O157:H7 (together or separately) or PBS and suspended in ADAMTS13-deficient plasma. Samples were perfused over glomerular endothelial cells. **A)** Hemolysis (OD405 nm) in plasma samples incubated with Shiga toxin 2 and O157LPS, PBS, Shiga toxin 2 alone or O157LPS alone. **B)** Arginase activity in plasma samples incubated with Shiga toxin 2 and O157LPS, PBS, Shiga toxin 2 alone or O157LPS alone. **C)** Arginase 1 concentration in lysates from red blood cells and primary glomerular endothelial cells, normalized to total protein concentration. Arginase 1 concentration in primary glomerular endothelial cells was below the detection limit of the assay. **D)** Healthy donor whole blood preincubated with Shiga toxin 2 and O157LPS or PBS was perfused over primary glomerular endothelial cells as above. No difference in hemolysis was noted. **E)** Arginase activity in the same healthy donor samples as in D. ADAMTS13: A Disintegrin and Metalloproteinase with a ThromboSpondin type 1 motif, member 13; O157LPS: Lipopolysaccharide from *E. coli* O157:H7; PBS: phosphate-buffered saline; PGEC: Primary glomerular endothelial cell; RBC: red blood cell; Stx2: Shiga toxin 2.

## Data Availability

The data included herein are reported within the published article and its supplementary information, and available from the corresponding author on request.

## Abbreviations

ADAMTS13: A Disintegrin And Metalloproteinase with a ThromboSpondin 1 motif, member 13
A1M: Alpha-1-microglobulin
DAPI: 4',6-Diamidino-2-phenylindole
EHEC: Enterohemorrhagic *Escherichia coli*
EHEC-HUS: Enterohemorrhagic *Escherichia coli*-associated hemolytic uremic syndrome
eNOS: Endothelial nitric oxide synthase
HUS: Hemolytic uremic syndrome
LDH: Lactate dehydrogenase
NO: Nitric oxide
NOS: Nitric oxide synthase
O157LPS: *E. coli* O157 lipopolysaccharide
OD: Optical density
PBS: Phosphate-buffered saline
PD: Peritoneal dialysis
PGEC: Primary glomerular endothelial cell
RBC: Red blood cell
Stx2: Shiga toxin 2
TMA: Thrombotic microangiopathy
TTP: Thrombotic thrombocytopenic purpura
