# Supplementary material for: Red blood cell-derived arginase release in hemolytic uremic syndrome
Source: J Transl Med. 2024 Jan 4;22:17. doi: 10.1186/s12967-023-04824-x (PMC10765883; doi:10.1186/s12967-023-04824-x)
Supplement: Supplementary file 1 — Additional file 1. Statistical comparisons performed in this study (Table). [file 12967_2023_4824_MOESM1_ESM.docx]

**Additional File 1: Statistical comparisons performed in this study**

| **Variable(s)** | **Cohort/model** | **Comparison (n)** | **Medians** | **Test** | **P value** | **Effect size estimate** | | | **Linear regression slope (95% CI)** | **Figure** |
| --- | --- | --- | --- | --- | --- | --- | --- | --- | --- | --- |
|  |  |  |  |  |  | Cohen’s r | PI (95% CI) | MDL (95% CI) |  |  |
| Arginase 1 | Cohort 1^a^ | Acute (23) vs controls (8) | 18.7 vs 5.73 ng/mL | KWD | 0.023 | 0.48 |  |  |  | 1A |
| Arginase 1 |  | Acute (23) vs remission (12) | 18.7 vs 2.62 ng/mL | KWD | 0.00037 | 0.65 |  |  |  | 1A |
| Arginase activity |  | Acute (24) vs controls (8) | 4.74 vs 1.09 µmol urea/(L*min) | KWD | 0.0039 | 0.57 |  |  |  | 1B |
| Arginase activity |  | Acute (24) vs remission (12) | 4.74 vs 0.733 µmol urea/(L*min) | KWD | <0.0001 | 0.75 |  |  |  | 1B |
| Arginase 1 |  | PD+ (15) vs PD- (8) | 27.9 vs 12.7 ng/mL | MWU | 0.034 |  | 0.78 (0.52-0.91) |  |  | 1C |
| Arginase activity |  | PD+ (15) vs PD- (9) | 5.25 vs 2.85 µmol urea/(L*min) | MWU | 0.015 |  | 0.80 (0.55-0.92) |  |  | 1D |
| Arginase 1 | Cohort 2^b^ | EHEC-HUS (23) vs controls (26) | 47.1 vs 10.4 ng/mL | MWU | <0.0001 |  | 0.88 (0.74-0.95) |  |  | 1E |
| Arginase 1, LDH | Cohort 1 | Arg1 vs LDH (22) |  | SLR | 0.017 |  |  |  | 0.25 (0.048-0.45), R^2^=0.25 | 1F |
| A1M |  | Acute (21) vs controls (8) | 30.7 vs 11.3 µg/mL | KWD | <0.0001 | 0.80 |  |  |  | 1G |
| A1M |  | Acute (21) vs remission (10) | 30.7 vs 15.7 µg/mL | KWD | 0.063 | 0.54 |  |  |  | 1G |
| Platelet count | EHEC mouse model | EHEC (13) vs PBS (8) | 486 vs 662 *10^9^/L | MWU | 0.045 |  | 0.77 (0.50-0.91) |  |  | 2C |
| Neutrophil count |  | EHEC (13) vs PBS (8) | 5.0 vs 1.3 *10^9^/L | MWU | <0.0001 |  | 0.98 (0.76-1.00) |  |  | 2D |
| Urea |  | EHEC (10) vs PBS (8) | 285 vs 60.7 mg/dL | MWU | <0.0001 |  | 0.99 (0.75-1.00) |  |  | 2E |
| Arginase 1 |  | EHEC (10) vs PBS (8) | 222 vs 12.4 ng/mL | MWU | 0.00021 |  | 0.99 (0.73-1.00) |  |  | 3A |
| Arginase activity |  | EHEC (10) vs PBS (8) | 136 vs 26.1 µmol urea/(L*min) | MWU | 0.0062 |  | 0.88 (0.60-0.97) |  |  | 3B |
| LDH |  | EHEC (10) vs PBS (8) | 31.4 vs 11.7 µkat/L | MWU | 0.0021 |  | 0.83 (0.54-0.95) |  |  | 3C |
| A1M |  | EHEC (10) vs PBS (8) | 12.2 vs 4.48 µg/mL | MWU | 0.0002 |  | 0.98 (0.73-1.00) |  |  | 3D |
| Arginase 1, LDH |  | Arg1 vs LDH (17) |  | SLR | 0.0044 |  |  |  | 1.21 (0.44-1.97), R^2^=0.43 | 3E |
| A1M |  | A1M vs arg1 (17) |  | SLR | 0.0013 |  |  |  | 0.23 (0.10-0.35), R^2^=0.51 | 3F |
|  |  | A1M vs LDH (18) |  | SLR | 0.010 |  |  |  | 0.35 (0.0.096-0.61), R^2^=0.35 | 3G |
| Urea | Stx2 mouse model | Stx2 (12) vs PBS (6) | 203 vs 44.5 mg/dL | MWU | 0.10 |  | 0.75 (0.45-0.91) |  |  | 4D |
| Arginase 1 |  | Stx2 (12) vs PBS (6) | 53.7 vs 14.3 ng/mL | MWU | 0.013 |  | 0.86 (0.57-0.96) |  |  | 4E |
| Arginase activity |  | Stx2 (12) vs PBS (6) | 23.5 vs 8.27 µmol urea/(L*min) | MWU | 0.0052 |  | 0.88 (0.61-0.97) |  |  | 4F |
| Hemolysis | *In vitro* TMA model^c^ | Stx (10) vs PBS (10) |  | WSR | 0.014 |  |  | 0.13  (0.046-0.22) |  | 5A |
| Arginase 1 |  | Stx (10) vs PBS (10) |  | WSR | 0.049 |  |  | 9.5 ng/mL  (1.1-19) |  | 5B |
| Arginase activity |  | Stx (10) vs PBS (10) |  | WSR | 0.0039 |  |  | 0.95 µmol urea/(L*min) (0.41-3.1) |  | 5C |
| Arginase 1, hemolysis |  | Arginase 1 vs hemolysis (10) |  | SLR | 0.0002 |  |  |  | 155 (99.2-212), R^2^=0.84 | 5D |

A1M: Alpha-1-microglobulin; CI: confidence interval; EHEC: Enterohemorrhagic *Escherichia coli*; KWD: Kruskal-Wallis test with Dunn’s multiple comparisons test; LDH: Lactate dehydrogenase activity; MDL: Median difference in location; MWU: Mann-Whitney U test; PBS: Phosphate-buffered saline; PD: Peritoneal dialysis; PI: Probabilistic index; SLR: Simple linear regression; Stx2: Shiga toxin 2; TMA: Thrombotic microangiopathy; WSR: Wilcoxon signed rank test. ^a^Cohort 1: Lund HUS cohort. ^b^Cohort 2: Philadelphia HUS cohort. ^c^Samples preincubated with Shiga toxin 2 and lipopolysaccharide from *Escherichia coli* O157:H7.
