# Supplementary material for: Red blood cell-derived arginase release in hemolytic uremic syndrome
Source: J Transl Med. 2024 Jan 4;22:17. doi: 10.1186/s12967-023-04824-x (PMC10765883; doi:10.1186/s12967-023-04824-x)
Supplement: Supplementary file 2 — Additional file 2. Correlation of arginase and urea in two mouse models. A) Plasma urea correlated with plasma arginase 1 in mice inoculated with E. coli O157:H7 or vehicle controls (n=17). B) Plasma urea correlated with plasma arginase activity in mice inoculated with E. coli O157:H7 or vehicle controls (n=17). C) Plasma urea correlated with plasma arginase 1 in mice injected with Shiga toxin 2 or vehicle controls (n=18). D) Plasma urea correlated with plasma arginase activity in mice injected with Shiga toxin 2 or vehicle controls (n=18). Comparisons performed using simple linear regression. [file 12967_2023_4824_MOESM2_ESM.pdf]

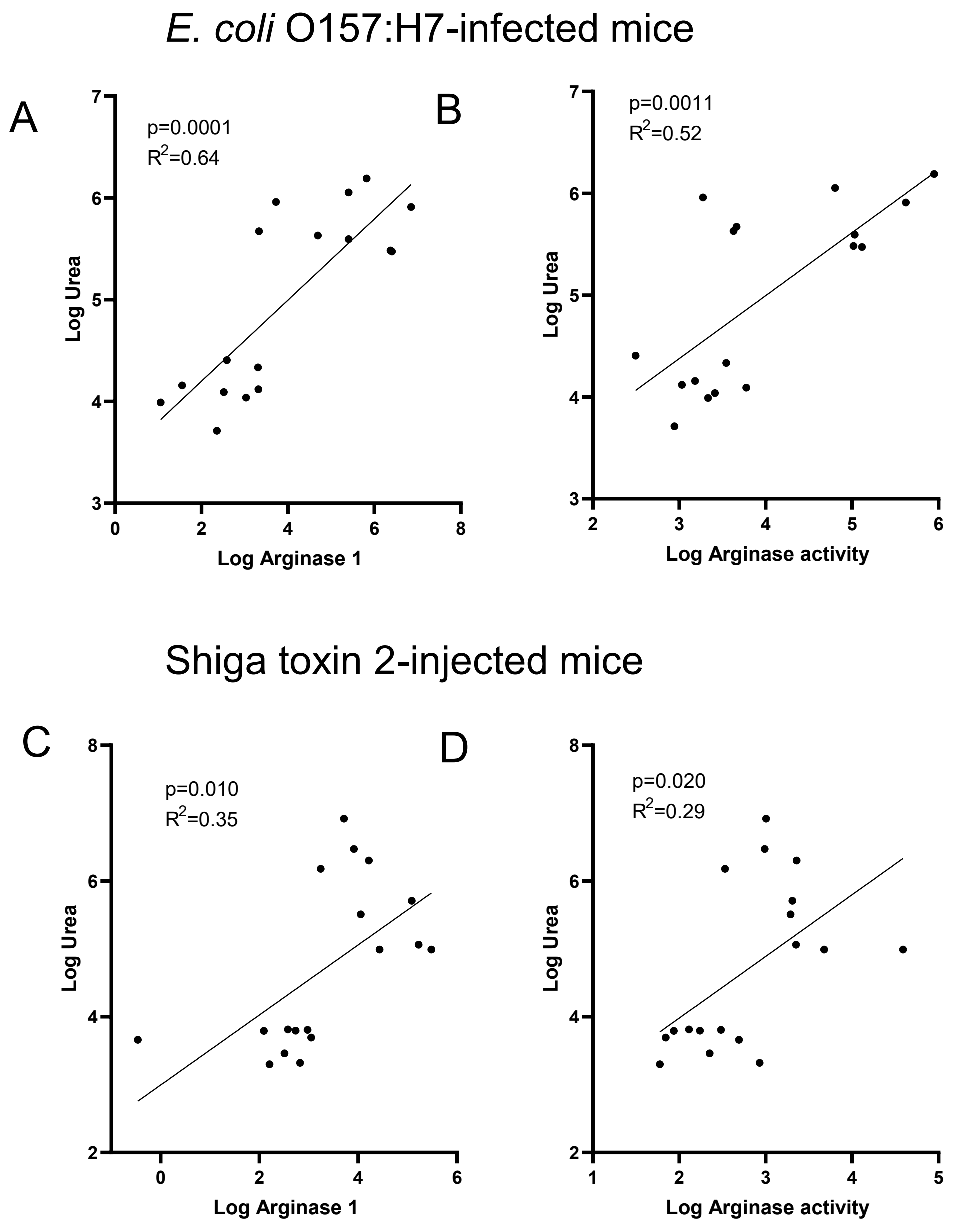

Additional File 2: Correlation of arginase and urea in two mouse models

A) Plasma urea correlated with plasma arginase 1 in mice inoculated with *E. coli* O157:H7 or vehicle controls (n=17). **B)** Plasma urea correlated with plasma arginase activity in mice inoculated with *E. coli* O157:H7 or vehicle controls (n=17). **C)** Plasma urea correlated with plasma arginase 1 in mice injected with Shiga toxin 2 or vehicle controls (n=18). **D)** Plasma urea correlated with plasma arginase activity in mice injected with Shiga toxin 2 or vehicle controls (n=18). Comparisons performed using simple linear regression.
