# Supplementary material for: Red blood cell-derived arginase release in hemolytic uremic syndrome
Source: J Transl Med. 2024 Jan 4;22:17. doi: 10.1186/s12967-023-04824-x (PMC10765883; doi:10.1186/s12967-023-04824-x)
Supplement: Supplementary file 3 — Additional file 3. Hemolysis and arginase in an in vitro model of thrombotic microangiopathy. Blood cells were pre-incubated with Shiga toxin 2 and/or lipopolysaccharide from E. coli O157:H7 (together or separately) or PBS and suspended in ADAMTS13-deficient plasma. Samples were perfused over glomerular endothelial cells. A) Hemolysis (OD405 nm) in plasma samples incubated with Shiga toxin 2 and O157LPS, PBS, Shiga toxin 2 alone or O157LPS alone. B) Arginase activity in plasma samples incubated with Shiga toxin 2 and O157LPS, PBS, Shiga toxin 2 alone or O157LPS alone. C) Arginase 1 concentration in lysates from red blood cells and primary glomerular endothelial cells, normalized to total protein concentration. Arginase 1 concentration in primary glomerular endothelial cells was below the detection limit of the assay. D) Healthy donor whole blood preincubated with Shiga toxin 2 and O157LPS or PBS was perfused over primary glomerular endothelial cells as above. No difference in hemolysis was noted. E) Arginase activity in the same healthy donor samples as in D. ADAMTS13: A Disintegrin and Metalloproteinase with a ThromboSpondin type 1 motif, member 13; O157LPS: Lipopolysaccharide from E. coli O157:H7; PBS: phosphate-buffered saline; PGEC: Primary glomerular endothelial cell; RBC: red blood cell; Stx2: Shiga toxin 2. [file 12967_2023_4824_MOESM3_ESM.pdf]

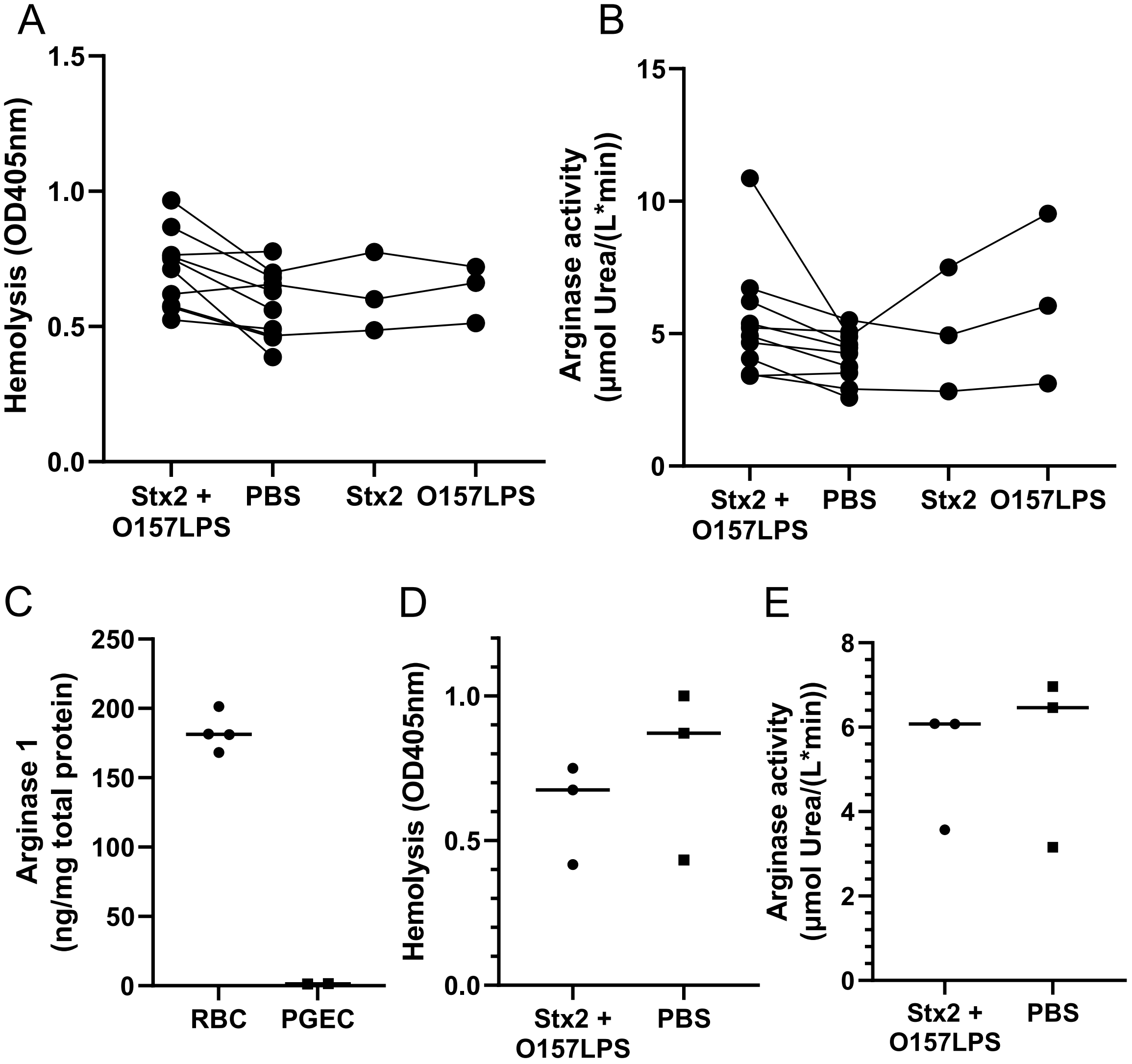

Additional File 3: Hemolysis and arginase in an *in vitro* model of thrombotic microangiopathy

Blood cells were pre-incubated with Shiga toxin 2 and/or lipopolysaccharide from *E. coli* O157:H7 (together or separately) or PBS and suspended in ADAMTS13-deficient plasma. Samples were perfused over glomerular endothelial cells. **A)** Hemolysis (OD405 nm) in plasma samples incubated with Shiga toxin 2 and O157LPS, PBS, Shiga toxin 2 alone or O157LPS alone. **B)** Arginase activity in plasma samples incubated with Shiga toxin 2 and O157LPS, PBS, Shiga toxin 2 alone or O157LPS alone. **C)** Arginase 1 concentration in lysates from red blood cells and primary glomerular endothelial cells, normalized to total protein concentration. Arginase 1 concentration in primary glomerular endothelial cells was below the detection limit of the assay. **D)** Healthy donor whole blood preincubated with Shiga toxin 2 and O157LPS or PBS was perfused over primary glomerular endothelial cells as above. No difference in hemolysis was noted. **E)** Arginase activity in the same healthy donor samples as in D. ADAMTS13: A Disintegrin and Metalloproteinase with a ThromboSpondin type 1 motif, member 13; O157LPS: Lipopolysaccharide from *E. coli* O157:H7; PBS: phosphate-buffered saline; PGEC: Primary glomerular endothelial cell; RBC: red blood cell; Stx2: Shiga toxin 2.
